# Sex disparities in the association between acute myocardial infarction and colon cancer risk

**DOI:** 10.1002/cam4.5205

**Published:** 2022-09-07

**Authors:** Shing‐Hsien Chou, Chia‐Pin Lin, Yu‐Sheng Lin, Ting‐Hein Lee, Chan‐Keng Yang, Yu‐Sheng Lin, Pao‐Hsien Chu

**Affiliations:** ^1^ Division of Cardiology, Linko Branch Chang Gung Memorial Hospital Taoyuan Taiwan; ^2^ Graduate Institute of Clinical Medical Sciences, College of Medicine Chang Gung University Taoyuan Taiwan; ^3^ Division of Cardiology, Chiayi Branch Chiayi Chang Gung Memorial Hospital Chiayi Taiwan; ^4^ Department of Anatomy, College of Medicine Chang Gung University Taoyuan Taiwan; ^5^ Division of Hematology‐Oncology Chang Gung Memorial Hospital Taoyuan Taiwan; ^6^ Healthcare center Chang Gung Memorial Hospital Taoyuan Taiwan; ^7^ Division of cardiology Department of Internal Medicine Chang Gung Memorial Hospital Taoyuan Branch Taoyuan Taiwan

**Keywords:** acute myocardial infarction (AMI), cardiovascular disease (CVD), colon cancer, inverse probability of treatment weighting (IPTW), sex disparity

## Abstract

**Background:**

Acute myocardial infarction (AMI) and colon cancer share similar risk factors. Studies have suggested an association between AMI and colon cancer; however, evidence is conflicting. Whether sex disparities exist in this association in the real world remains unknown.

**Methods:**

In this population‐based retrospective cohort study, 94,780 and 97,987 male patients and 38,697 and 72,007 female patients with and without new‐onset AMI, respectively, from January 1, 2001, to December 31, 2012, were enrolled from Taiwan's National Health Insurance Research Database. Inverse probability of treatment weighting (IPTW) was used to balance covariates across study groups. The primary outcome was a new diagnosis of colon cancer.

**Results:**

The incidence rate of colon cancer was 1.54 (95% confidence interval [CI] = 1.46–1.62) and 1.40 (95% CI = 1.32–1.48) per 1000 person‐years in the male patients and 1.62 (95% CI = 1.50–1.74) and 1.22 (95% CI = 1.13–1.32) in the female patients, in the AMI and non‐AMI groups, respectively. AMI was associated with a significantly higher risk of colon cancer in the female patients (hazard ratio [HR] = 1.31, 95% CI = 1.06–1.61) but not in the male patients (HR = 1.09, 95% CI = 0.95–1.26). In the subgroup analysis, the association between AMI and colon cancer in the female patients was stronger in those aged ≥65 years (HR = 1.28, 95% CI = 1.13–1.44).

**Conclusions:**

An increased risk of colon cancer was observed only in the female patients with AMI. The association between AMI and colon cancer in the female patients was the most evident in those aged ≥65 years.

## Graphical abstract

This study is one of the largest studies to examined the risk of colon cancers in patients with AMI. The results showed that AMI was associated with a significantly higher risk of colon cancer in the female patients, but not in the male patients. Cardiologists who treat patients with acute myocardial infarction should be aware of colon cancer risk, especially in women.

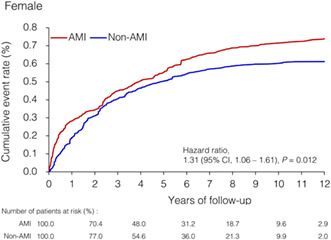

## 1 INTRODUCTION

Acute myocardial infarction (AMI) and colon cancer are two of the most common diseases and leading causes of deaths worldwide. Approximately 9 million deaths were attributed to ischemic heart disease in 2017 globally, including AMI.^1^ Colorectal cancer (CRC) is the third most common cancer and accounted for 10% of new cancer cases and 9.4% of cancer deaths globally in 2020.^2^ Although AMI and other atherosclerotic cardiovascular disease (CVD) are distinct from colon cancer, they share several common risk factors and biological mechanisms.^3^, ^4^, ^5^

Epidemiological studies have explored the association of CVD with the incidence of advanced colorectal neoplasms.^6^, ^7^, ^8^, ^9^ Chan et al. reported a higher risk of advanced colorectal neoplasms (odds ratio [OR]: 2.51) in patients with newly diagnosed coronary artery disease (CAD).^6^ High CVD risk, as indicated by a Framingham risk score (FRS) in the high‐risk tertile, was linked to increased ORs for advanced colorectal neoplasm (OR: 3.83).^8^ However, whether this association holds between AMI and colon cancer, the most severe forms of CAD and colon neoplasms, respectively, especially after adjustment for traditional cardiovascular risk factors, remains unclear.

Differences between male and female were noted regarding the incidence rate, risk profiles, and biological features of AMI and colon cancer. These inherent differences may exert a distinct effect on the association of AMI and colon cancer in different sexes. Women have lower incidences of CAD and AMI than did age‐matched men,^10^ especially in the premenopausal period. In addition, the Global Cancer Observatory also reported lower prevalence of CRC in women after standardization by age.^11^ Furthermore, the profiles of risk factors are somewhat different between men and women. Diabetes mellitus (DM), hypertension, and physical inactivity were reported to be more strongly associated with AMI in women, whereas cigarette smoking and dyslipidemia were less prevalent in women.^12^ Significant biological differences in sex hormones and genes are observed between men and women, and these differences can considerably affect the initiation and progression of both AMI^13^ and colon cancer.^14^, ^15^ Whether sex disparities exert a different effect on the association between AMI and colon cancer, and potentially influence the strategies to screen and prevent colon cancer in AMI survivors in different sexes, merits further research. To our knowledge, no study has examined this topic. Therefore, in the current study, we investigated the risk of colon cancer in patients with AMI as well as the possibility of sex disparities in the association between AMI and colon cancer.

## 2 MATERIALS AND METHODS

### 2.1 Data source

This nationwide retrospective cohort study was performed using data from the National Health Insurance (NHI) Research Database (NHIRD) and Longitudinal Health Insurance Database 2000 (LHID2000). The NHI is Taiwan's single‐payer health insurance system covering >99% of the population. The large database derived from the NHIRD contains registration files, detailed medical data, and original claim data from reimbursement files. The LHID2000 contains all the original claim data of 1000,000 individuals randomly sampled from the Registry for Beneficiaries of the NHIRD for the year 2000.^16^ Because the original identification number of all patients in the NHIRD and LHID2000 is encrypted and deidentified by using a consistent encrypting procedure, the requirement of informed consent was waived. The study protocol was approved by the Institutional Review Board of Chang Gung Memorial Hospital (201800819B0).

### 2.2 Study design

**FIGURE 1:**
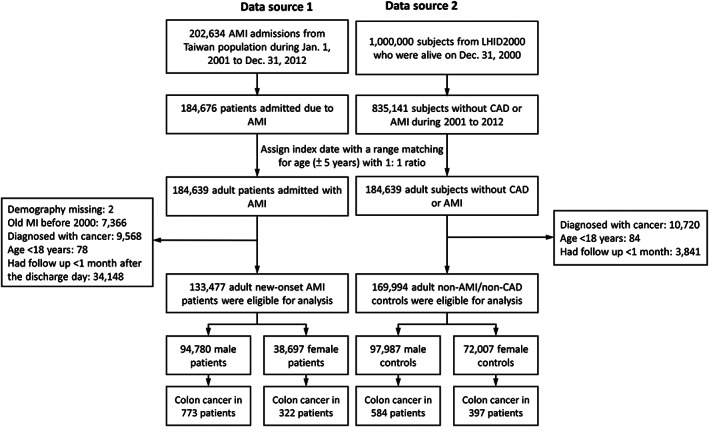
Flowchart for the inclusion and exclusion of patients with AMI and those without AMI. AMI, acute myocardial infarction; CAD, coronary arterial disease; LHID 2000, Longitudinal Health Insurance Database 2000.

A flowchart of study cohort enrollment is presented in Figure 1. Totally 202,634 AMI admissions (contributed by 184,676 patients) between January 1, 2001, and December 31, 2012, were identified from the NHIRD (data source 1). For the AMI group, the discharge day from the index AMI admission was defined as the index date. Totally 835,141 patients without a history of CAD or AMI were identified from the LHID2000 (data source 2). After performing a range matching for age (±5 years) at a 1:1 ratio to data source 1 and assigning the index date corresponding to the matching patients in data source 1, we identified 184,639 matching patients without CAD or AMI in data source 2 and 184,639 patients with AMI in data source 1. The approach of assigning the index date of the AMI patient to the corresponding non‐AMI subject was known as “prescription time distribution matching” to avoid the immortal time bias.^17^

Among the 184,639 patients with AMI, two were excluded because of missing demographic data. In addition, we excluded patients who experienced myocardial infarction before December 31, 2000 (*n* = 7366), received a diagnosis of cancer before the index date (*n* = 9568), or were aged <18 years (*n* = 78). Patients who had a follow‐up duration of <1 month after discharge for the index AMI admission (*n* = 34,148) were also excluded. This criterion was used to exclude the patients who died during hospitalization or died very early after discharge, as these patients would not have the opportunity to develop colon cancer after the index AMI. In total, 133,477 adult patients with new‐onset AMI were eligible for this study (AMI group), including 94,780 male patients and 38,697 female patients.

Among the 184,639 patients without CAD and AMI, those who received a diagnosis of cancer before the index date (*n* = 10,720), were aged <18 years (*n* = 84), or had a follow‐up duration of <1 month (*n* = 3841) after the index date were excluded. In total, 169,994 adult patients without CAD and AMI were eligible for this study as the control group (non‐AMI group), including 97,987 male patients and 72,007 female patients. The follow‐up period was defined as the duration from the index date until the occurrence of the study outcome, death, or the end date of the study period (December 31, 2012), whichever occurred earlier.

### 2.3 Exposure

Patients with AMI were identified as those who received a principal diagnosis of AMI (*International Classification of Diseases, Ninth Revision, Clinical Modification* [*ICD‐9‐CM*] code 410.xx) during discharge from hospitalization. A study validating the use of *ICD‐9‐CM* codes for identifying AMI reported a positive predictive value of 0.92 and a sensitivity of 0.88.^18^ Patients who did not develop AMI or CAD during the study period were included in the non‐AMI group. This criterion minimized the possibility of including in this group patients who had CAD but did not develop AMI.

### 2.4 Study outcome

The primary outcome of this study was a new diagnosis of colon cancer during the study period. In Taiwan, an identification card for catastrophic illnesses is issued to patients with major illnesses including any malignancy. To prevent misclassification, the diagnosis of colon cancer was based on the presence of the *ICD‐9‐CM* code 153.xx with the ascertainment of possessing an identification card for catastrophic illnesses because of colon cancer.

### 2.5 Covariates

Baseline covariates were identified according to relevant claim records with diagnoses or medication codes before the index date. The urbanization level of the patient's place of residence was categorized as low, moderate, high, or very high,^19^ and was used as a proxy for socioeconomic status. Several comorbidities were also extracted, including hypertension, DM, dyslipidemia, atrial fibrillation, and chronic kidney disease. The presence of comorbidity was defined as having ≥2 outpatient diagnoses or any one inpatient diagnosis in the previous year.

### 2.6 Statistical analysis

To make a comparability between the AMI patients and non‐AMI matched controls when comparing the risk of colon cancer, an additional cohort was created by the inverse probability of treatment weighting (IPTW) based on propensity score. The propensity score was the predicted probability to be in the AMI group given certain values of covariates derived from the multivariable logistic regression model. To avoid the impact of extreme weights, we obtained the stabilized weights and truncated the extreme weights at the 99th percentile. The balance of baseline demographics and characteristics between the AMI patients and non‐AMI matched controls were assessed by using standardized difference (STD), of which an absolute value less than 0.2 was considered non‐substantial difference between groups.

The incidence of colon cancer was expressed as the incidence density, which was the number of events per 1000 person years. The risk of colon cancer between the AMI patients and non‐AMI matched controls was compared using the Cox proportional hazard model. The primary analysis was further stratified by several pre‐specified subgroup variables: age, DM, statin use at baseline, and the urbanization level. A significant interaction in the subgroup analysis indicated that the association between AMI and colon cancer risk was modified by the subgroup variable. The study groups (AMI vs. non‐AMI) were the only explanatory factor in the aforementioned survival analyses. All the analyses as well as IPTW were conducted for men and women separately. A two‐sided *p* value <0.05 was considered to be statistically significant. All analyses were conducted with SAS software of version 9.4 (SAS Institute Inc.).

## 3 RESULTS

This study enrolled a total of 94,780 and 97,987 consecutive male patients and 38,697 and 72,007 consecutive female patients with and without new‐onset AMI, respectively, from January 1, 2001, to December 31, 2012. Colon cancer was diagnosed in 773 (0.82%) and 584 (0.60%) male patients and 332 (0.83%) and 397 (0.55%) female patients in the AMI and non‐AMI groups, respectively (Figure 1). Before IPTW, the patients in the AMI group were older and had more comorbidities than did those in the non‐AMI group in both the male (Table 1) and female cohorts (Table 2). After IPTW adjustment, all of the characteristics were well‐balanced (absolute value of STD <0.2) between the AMI and non‐AMI groups in the male and female cohorts. The mean follow‐up durations in the AMI and non‐AMI groups were 5.0 and 4.9 years, respectively, in the male cohort and 4.6 and 5.0 years, respectively, in the female cohort.

**TABLE 1:** Demographics and characteristics of male patients with AMI and those without AMI (*N* = 192,767)

	Before IPTW^a^			After IPTW^b^		
Variable	AMI (*n* = 94,780)	Non‐AMI (*n* = 97,987)	STD	AMI	Non‐AMI	STD
Age, years	63.2 ± 13.9	60.0 ± 12.6	0.24	62.1 ± 14.0	62.4 ± 12.9	−0.02
Age group						
<50 years	17,670 (18.6)	19,982 (20.4)	−0.04	18.7	16.8	0.05
50–64 years	33,512 (35.4)	46,660 (47.6)	−0.25	40.1	42.0	−0.04
≥65 years	43,598 (46.0)	31,345 (32.0)	0.29	41.2	41.2	<0.01
Urbanization level						
Low	10,913 (11.5)	11,399 (11.6)	<0.01	11.7	11.6	<0.01
Moderate	29,489 (31.1)	31,004 (31.6)	−0.01	31.4	31.5	<0.01
High	28,240 (29.8)	28,728 (29.3)	0.01	29.5	29.5	<0.01
Very High	26,138 (27.6)	26,856 (27.4)	<0.01	27.4	27.4	<0.01
Region						
North	38,650 (40.8)	38,775 (39.6)	0.02	40.0	39.6	0.01
Central	21,657 (22.8)	24,268 (24.8)	−0.05	23.6	23.9	−0.01
South	31,129 (32.8)	31,342 (32.0)	0.02	32.7	32.7	<0.01
East	3344 (3.5)	3602 (3.7)	−0.01	3.7	3.7	<0.01
Comorbidities						
Hypertension	57,436 (60.6)	20,735 (21.2)	0.88	43.0	39.4	0.07
Diabetes mellitus	32,501 (34.3)	10,528 (10.7)	0.59	24.4	23.6	0.02
Dyslipidemia	36,169 (38.2)	7757 (7.9)	0.77	24.7	19.2	0.13
Atrial fibrillation	5895 (6.2)	578 (0.6)	0.31	3.5	2.8	0.04
Chronic kidney disease	16,209 (17.1)	3624 (3.7)	0.45	11.0	10.6	0.01
Follow‐up year	4.9 ± 3.4	5.2 ± 3.4	−0.10	5.0 ± 3.5	4.9 ± 3.4	0.03

Abbreviations: AMI, acute myocardial infarction; IPTW, inverse probability treatment weighting; STD, standardized difference.

^a^ Data are presented as the frequency (percentage) or the mean ± standard deviation.

^b^ Data are presented as the percentage or the mean ± standard deviation.

**TABLE 2:** Demographics and characteristics of female patients with AMI and those without AMI (*N* = 110,704)

	Before IPTW^a^			After IPTW^b^		
Variable	AMI (*n* = 38,697)	Non‐AMI (*n* = 72,007)	STD	AMI	Non‐AMI	STD
Age, years	71.6 ± 12.4	64.4 ± 11.1	0.62	68.1 ± 13.4	67.3 ± 11.7	0.07
Age group						
<50 years	2283 (5.9)	3904 (5.4)	0.02	7.0	5.0	0.09
50–64 years	8080 (20.9)	37,472 (52.0)	−0.68	32.7	40.1	−0.16
≥65 years	28,334 (73.2)	30,631 (42.5)	0.65	60.3	54.9	0.11
Urbanization level						
Low	6027 (15.6)	8698 (12.1)	0.10	14.0	13.2	0.02
Moderate	12,473 (32.2)	21,880 (30.4)	0.04	31.9	31.2	0.02
High	10,768 (27.8)	21,091 (29.3)	−0.03	28.4	28.8	−0.01
Very High	9429 (24.4)	20,338 (28.2)	−0.09	25.6	26.9	−0.03
Region						
North	14,531 (37.6)	28,909 (40.1)	−0.05	38.1	39.0	−0.02
Central	8976 (23.2)	17,345 (24.1)	−0.02	23.6	23.7	<0.01
South	13,459 (34.8)	23,182 (32.2)	0.05	34.0	33.4	0.01
East	1731 (4.5)	2571 (3.6)	0.05	4.3	3.9	0.02
Comorbidities						
Hypertension	28,984 (74.9)	20,133 (28.0)	1.06	51.9	46.2	0.11
Diabetes mellitus	19,526 (50.5)	9661 (13.4)	0.87	32.3	29.0	0.07
Dyslipidemia	13,220 (34.2)	8534 (11.9)	0.55	23.9	21.4	0.06
Atrial fibrillation	3960 (10.2)	459 (0.6)	0.43	4.7	3.8	0.04
Chronic kidney disease	10,201 (26.4)	2527 (3.5)	0.68	13.7	11.9	0.06
Follow‐up year	4.1 ± 3.3	5.5 ± 3.3	−0.42	4.6 ± 3.5	5.0 ± 3.3	−0.13

Abbreviations: AMI, acute myocardial infarction; IPTW, inverse probability treatment weighting; STD, standardized difference.

^a^ Data are presented as the frequency (percentage) or the mean ± standard deviation.

^b^ Data are presented as the percentage or the mean ± standard deviation.

**FIGURE 2:**
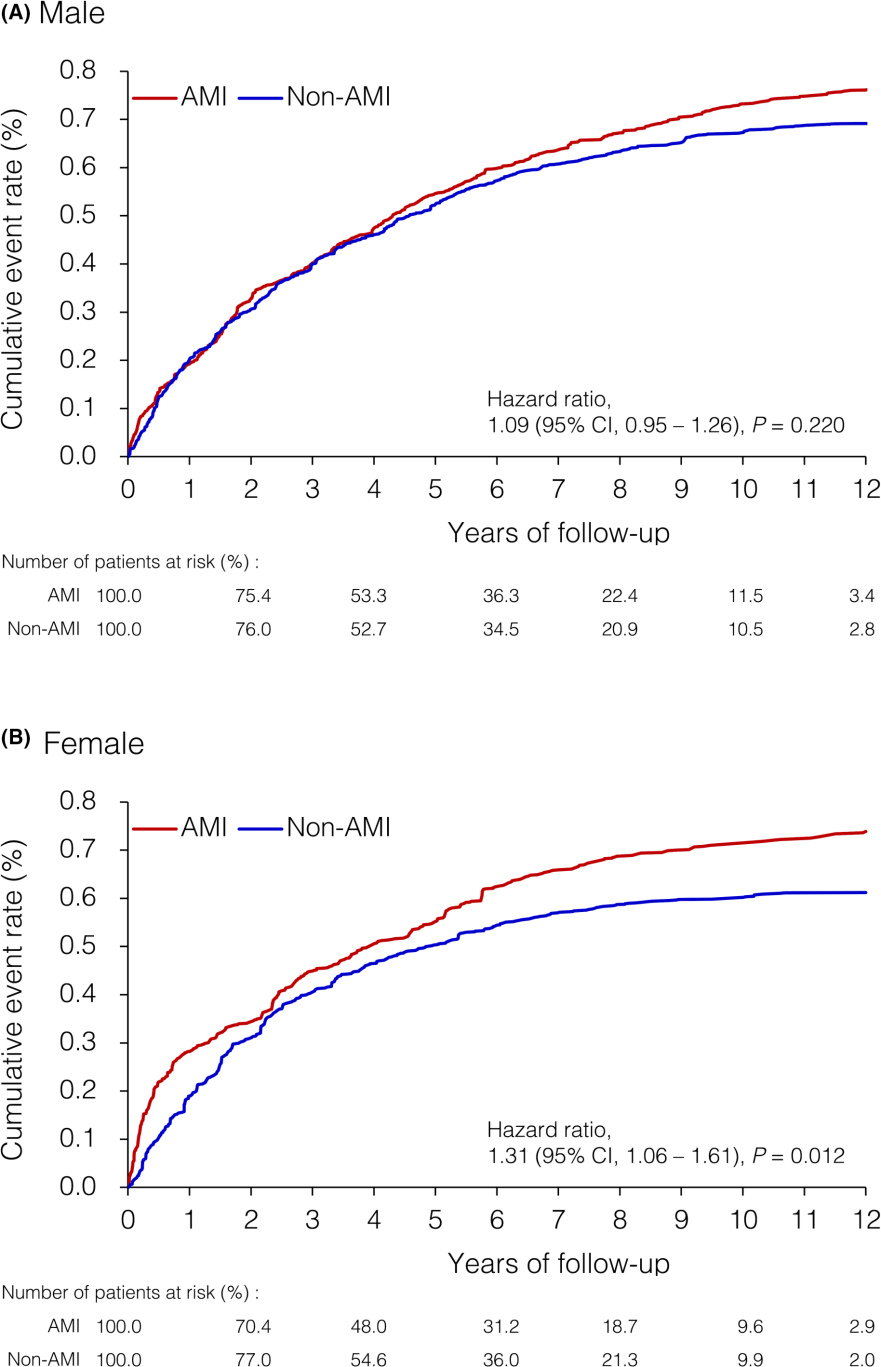
Cumulative event rate of colon cancer for the male (A) and female (B) patients with AMI and those without AMI in the IPTW‐adjusted cohorts. AMI, acute myocardial infarction; CI, confidence interval; IPTW, inverse probability of treatment weighting.

In the male cohort, the incidence rate of colon cancer after IPTW adjustment was 1.54 (95% confidence interval [CI] = 1.46–1.62) and 1.40 (95% CI = 1.32–1.48) per 1000 person‐years in the AMI and non‐AMI groups, respectively. No difference in the risk of colon cancer was observed between the AMI and non‐AMI groups (hazard ratio [HR] = 1.09, 95% CI = 0.95–1.26; Table 3). The cumulative event curve for colon cancer after IPTW adjustment did not significantly differ between the groups (Figure 2A).

**TABLE 3:** Association between AMI and colon cancer risk in male patients after IPTW adjustment

Subgroup	Incidence (95% CI)^a^		HR (95% CI) for AMI	*p* for interaction
	AMI	Non‐AMI		
Total	1.54 (1.46–1.62)	1.40 (1.32–1.48)	1.09 (0.95–1.26)	—
Age				0.253
<50 years	0.27 (0.20–0.34)	0.34 (0.26–0.42)	0.79 (0.55–1.14)	
50–64 years	1.17 (1.06–1.27)	1.07 (0.96–1.17)	1.08 (0.95–1.23)	
≥65 years	2.88 (2.69–3.07)	2.66 (2.46–2.85)	1.09 (0.98–1.20)	
Diabetes mellitus				0.208
No	1.39 (1.30–1.47)	1.23 (1.15–1.31)	1.13 (1.03–1.23)	
Yes	2.24 (2.01–2.48)	2.23 (1.99–2.47)	1.01 (0.87–1.17)	
Urbanization level				0.033
Low/Moderate	1.48 (1.36–1.60)	1.22 (1.11–1.34)	1.21 (1.07–1.37)	
High/Very high	1.58 (1.47–1.68)	1.54 (1.43–1.64)	1.02 (0.93–1.13)	
Statin user				0.003
No	1.44 (1.35–1.52)	1.38 (1.30–1.47)	1.04 (0.95–1.13)	
Yes	2.14 (1.89–2.39)	1.52 (1.31–1.74)	1.40 (1.17–1.68)	

Abbreviations: AMI, acute myocardial infarction; CI, confidence interval; HR, hazard ratio; IPTW, inverse probability treatment weighting.

^a^ Number of events per 1000 person‐years.

The findings of subgroup analysis were consistent across different prespecified age subgroups in the male cohort, with AMI not associated with a higher risk of colon cancer across these subgroups (Table 3). Because DM, socioeconomic status, or statin use at baseline might affect the incidence of colon cancer, we analyzed the effects of these covariates on AMI and colon cancer risk. Prominent heterogeneity was observed in the subgroups of the patients with different socioeconomic statuses and statin use at baseline (P for interaction <0.05). In those with lower socioeconomic status, as indicated by a low or moderate urbanization level, AMI was associated with an increased risk of colon cancer (HR = 1.21, 95% CI = 1.07–1.37) but not in those with higher socioeconomic status (HR = 1.02, 95% CI = 0.93–1.13). In addition, AMI was associated with a higher risk of colon cancer in those with statin use at baseline (HR = 1.40, 95% CI = 1.17–1.68) but not in those without statin use (HR = 1.04, 95% CI = 0.95–1.13) at baseline. DM status did not modify the effect of AMI on the risk of colon cancer (P for interaction >0.05).

In the female cohort, the incidence rate of colon cancer after IPTW adjustment was 1.62 (95% CI = 1.50–1.74) and 1.22 (95% CI = 1.13–1.32) per 1000 person‐years in the AMI and non‐AMI groups, respectively. AMI was associated with a significantly higher risk of colon cancer (HR = 1.31, 95% CI = 1.06–1.61; Table 4). Early separation of the cumulative event curve for colon cancer after IPTW adjustment was noted between the AMI and non‐AMI groups (Figure 2B).

**TABLE 4:** Association between AMI and colon cancer risk in female patients after IPTW adjustment

Subgroup	Incidence (95% CI)^a^		HR (95% CI) for AMI	*p* for interaction
	AMI	Non‐AMI		
Total	1.62 (1.50–1.74)	1.22 (1.13–1.32)	1.31 (1.06–1.61)	—
Age				0.320
<50 years	0.30 (0.13–0.47)	0.30 (0.14–0.46)	0.97 (0.44–2.10)	
50–64 years	0.79 (0.66–0.92)	0.73 (0.63–0.83)	1.07 (0.86–1.33)	
≥65 years	2.52 (2.31–2.73)	1.98 (1.80–2.16)	1.28 (1.13–1.44)	
Diabetes mellitus				<0.001
No	1.36 (1.23–1.48)	1.21 (1.11–1.31)	1.11 (0.98–1.26)	
Yes	2.43 (2.14–2.73)	1.28 (1.08–1.48)	1.88 (1.54–2.30)	
Urbanization level				0.828
Low/Moderate	1.57 (1.40–1.75)	1.18 (1.04–1.32)	1.33 (1.13–1.56)	
High/Very high	1.66 (1.49–1.82)	1.26 (1.14–1.38)	1.30 (1.13–1.49)	
Statin user				<0.001
No	1.36 (1.24–1.48)	1.22 (1.12–1.33)	1.10 (0.97–1.24)	
Yes	2.56 (2.24–2.89)	1.22 (1.03–1.42)	2.06 (1.67–2.53)	

Abbreviations: AMI, acute myocardial infarction; CI, confidence interval; HR, hazard ratio; IPTW, inverse probability treatment weighting.

^a^ Number of events per 1000 person‐years.

In the subgroup analysis, the association between AMI and higher colon cancer risk was the most evident in the patients aged ≥65 years (HR = 1.28, 95% CI = 1.13–1.44). This association was not observed in the patients aged <50 (HR = 0.97, 95% CI = 0.44–2.10) and 50–64 years (HR = 1.07, 95% CI = 0.86–1.33). In the subgroup analysis of DM status, socioeconomic status, or statin use at baseline, a consistently higher risk of colon cancer in the AMI versus non‐AMI group was observed across different socioeconomic statuses. However, this association was observed in the subgroups with DM (HR = 1.88, 95% CI = 1.54–2.30) or statin use (HR = 2.06, 95% CI = 1.67–2.53) at baseline, but not in those without DM (HR = 1.11, 95% CI = 0.98–1.26) or statin use (HR = 1.10, 95% CI = 0.97–1.24; P for interaction <0.05 in these subgroups) at baseline (Table 4).

## 4 DISCUSSION

This study investigated the risk of colon cancer after hospitalization for new‐onset AMI in the Asian population. Our study demonstrated sex disparities in the association between AMI and colon cancer. In the female cohort, the AMI group had a significantly higher risk of colon cancer than did the non‐AMI group. This association was the most evident in the patients aged ≥65 years. In the male cohort, no difference in the risk of colon cancer was observed between the AMI and non‐AMI groups.

Although previous studies have highlighted the association between CVD and colorectal neoplasms, the design of the current study is unique in several aspects. First, this is a large‐scale population‐based study enrolling 192,767 and 110,704 patients in the male and female cohorts, respectively, with a substantial number of patients in the cohorts developing colon cancer. Most of the previous studies have included the diagnosis of colorectal polyps, adenoma, or advanced neoplasm (defined as adenoma with the villous component, size ≥10 mm, with high‐grade dysplasia or cancer) as the main outcome measure.^6^, ^7^, ^8^, ^9^, ^20^, ^21^ In this study, the diagnosis of the most severe form of these lesions, colon cancer, was the primary outcome. The degree of CVD was evaluated on the basis of the FRS,^7^, ^8^, ^9^ presence of CAD,^6^, ^8^ or coronary computed tomography findings^20^, ^21^ in previous studies. To date, few studies have specifically evaluated the association between AMI and colon cancer.^22^, ^23^ Furthermore, except for age and sex, most of the shared risk factors between CVD and colorectal neoplasms were not sufficiently adjusted in previous studies. In this study, many of these risk factors were well‐balanced through IPTW adjustment, including age, socioeconomic status, DM, hypertension, and dyslipidemia.

Several studies have reported the association between CVD and an increased risk of colorectal neoplasm,^6^, ^7^, ^8^, ^9^, ^20^, ^21^ most likely due to shared risk factors and biological mechanisms between them.^3^, ^4^, ^5^ Traditional CVD risk factors, especially DM,^24^ obesity,^25^, ^26^ cigarette smoking,^27^ and physical inactivity,^28^, ^29^ have been linked to a higher risk of CRC. Consumption of processed meats, an established risk factor for CRC, was associated with a 42% increased risk of CVD.^30^ Underlying biological mechanisms, such as chronic inflammation and oxidative stress, that contribute to both CVD and CRC may drive these associations.^3^, ^31^ In our study, the association between AMI and colon cancer was still observed in the female cohort after IPTW adjustment for age, socioeconomic status, DM, hypertension, and dyslipidemia. This finding indicates that the association between AMI and colon cancer in the female cohort cannot be explained by these factors; however, the effects of imbalanced residual shared risk factors cannot be ruled out. Nevertheless, the differences in results between the female and male cohorts could not be explained by residual shared risk factors.

To our knowledge, this is the first study to demonstrate sex disparities in the association between AMI and colon cancer. A possible explanation for the sex‐specific association is the effect of estrogen and early‐onset menopause on both AMI and colon cancer in women. Emerging evidence indicates that estrogen may exert a protective effect on carcinomatosis in the colon. Women are less susceptible to CRC than men are, with the incidence rate being 31%–44% higher in men.^11^, ^32^ Exposure to sex hormones, especially estrogen, may partly explain this sex‐based difference. Hormone replacement therapy (HRT) may reduce CRC risk in postmenopausal women, with one study reporting an approximately 38% and 40% lower risk of colon and rectal cancer, respectively, in estrogen users.^33^ Furthermore, estrogen receptor β (ERβ) is the predominant estrogen receptor expressed in both the normal and malignant colonic epithelium.^34^ The expression of ERβ declines during colon cancer progression,^35^, ^36^ and this decreased expression is probably related to more advanced tumor stage, grade, and other characteristics of poor prognosis.^34^, ^36^ Epidemiological studies also suggested a protective role of estrogen against CVD. The incidence of CVD was considerably lower in women than in age‐matched men,^10^ especially in the premenopausal period. The occurrence of menopause was associated with an increased risk of CVD in women,^37^, ^38^ and young women who underwent bilateral oophorectomy had a higher risk of CAD.^39^ These findings imply the protective effect of estrogen on both colon cancer and CVD. Early‐onset menopause, which is characterized by a decline in estrogen level in earlier age, may lead to longer accumulation of adverse changes caused by decreased estrogen levels, including body fat distribution,^40^ lipid profile alterations,^41^ metabolic syndrome,^42^ and vascular remodeling.^43^ These changes in estrogen levels and CVD risk factors in midlife can contribute to CVD development later in life.^44^ Epidemiological studies also indicated that early‐onset menopause is linked to increased risks of both CAD^45^ and CRC.^46^ Decreased estrogen levels and early‐onset menopause may serve as shared risk factors for both AMI and colon cancer and at least partly explain the association between them in the female cohort in our study. Future investigations exploring this postulation are warranted.

In this study, the association between AMI and colon cancer in the female cohort was the most evident in the patients aged ≥ 65 years, although the results of subgroup analysis should not be overinterpreted. Patients aged ≥ 65 years constituted the largest portion of the female cohort in our study. This age group also accounted for approximately 58% of newly diagnosed colon cancer in the United States in 2020.^32^ CRC is uncommon before the age of 40 years, and only around 10% of cases occur before the age of 50 years.^32^ Risk factors for CRC likely differ between young and older adults. CRC in young adults is more likely related to inherited CRC syndrome^47^ or a history of CRC in a first‐degree relative,^48^ which may attenuate the effect of shared risk factors on the association between AMI and colon cancer. In addition, the longer accumulation of the adverse effects of shared risk factors in older women than in younger women possibly exert a stronger effect on the association between AMI and colon cancer in the former group. The results of our study do not imply that colon cancer risk in young patients with AMI merits less attention but rather highlight the importance of early screening and risk modification in midlife when prevention is most likely possible.

Our study has several strengths. We used a population‐based design within Taiwan's single‐payer health insurance system with a complete medical history. To our knowledge, our cohort of patients with AMI is the largest among studies examining this association in the Asian population. To prevent misclassification, the diagnosis of colon cancer based on *ICD‐9‐CM* codes was further confirmed by the issuance of an identification card for catastrophic illness. The diagnosis of AMI was based on the *ICD‐9‐CM* code at discharge, which was validated to have a high positive predictive value.^18^ We performed an age‐matched and sex‐separate study, and age, socioeconomic status, and confounding comorbidities were further adjusted using the IPTW method. These features reduced the risk of selection bias and minimized the confounding effects of these factors.

Our study has potential limitations. First, the status of cigarette smoking, obesity, physical inactivity, and diet cannot be obtained from the NHIRD database. These factors may serve as residual shared risk factors and have a role in this association. Second, the status of aspirin use was not balanced by IPTW in this study. Aspirin is generally prescribed to patients with AMI but not frequently prescribed to patients in the non‐AMI group; thus, we could not adjust for this factor. However, use of aspirin has been reported to have a protective role in CRC^49^, ^50^ and might have attenuated the association of colon cancer risk in the AMI group. Third, although the incidence rate of CRC is similar between Taiwan and Western population,^11^, ^51^ the incidence rate of AMI has been reported to be much lower in Taiwan.^52^, ^53^ It remained unclear whether our results can extend to the non‐Asian populations. Finally, the study period was from January 1, 2001, to December 31, 2012, with a mean follow‐up duration of 4.6–5.0 years. Additional studies with longer follow‐up periods can enable the evaluation of the effects of shared risk factors and mechanisms accumulated for a longer period and may provide greater insights into the association between AMI and colon cancer.

In conclusion, this study demonstrated sex disparities in the association between AMI and colon cancer risk. The female patients with AMI had a significantly increased risk of colon cancer, which was the most evident in those aged ≥65 years; however, no such association was noted in the male patients with AMI. Cardiologists who treat patients with AMI should be aware of colon cancer risk, especially in women. Colon cancer screening and modification of risk factors are suggested for female survivors with AMI to detect and prevent potentially treatable early colon cancer.

## AUTHOR CONTRIBUTIONS

**Shing‐Hsien Chou:** Conceptualization (lead); data curation (lead); formal analysis (lead); investigation (lead); methodology (lead); writing – original draft (lead); writing – review and editing (lead). **Chia‐Pin Lin:** Data curation (supporting); formal analysis (supporting); writing – review and editing (supporting). **Yu‐Sheng Lin:** Data curation (supporting); formal analysis (supporting); writing – review and editing (supporting). **Ting‐Hein Lee:** Data curation (supporting); formal analysis (supporting); writing – review and editing (supporting). **Chan‐Keng Yang:** Data curation (supporting); formal analysis (supporting); writing – review and editing (supporting). **Yu‐Sheng Lin:** Conceptualization (lead); data curation (lead); formal analysis (equal); funding acquisition (equal); writing – review and editing (equal). **Pao‐Hsien Chu:** Conceptualization (equal); data curation (equal); funding acquisition (equal).

## FUNDING INFORMATION

Chang Gung Memorial Hospital Taiwan (grant CMRPG5H0221).

## CONFLICT OF INTEREST

The authors declare no potential conflict of interests.

## Data Availability

The data used in the analysis are available from the corresponding author upon reasonable request.
